# Prenatal alcohol exposure and white matter microstructural changes across the first 6–7 years of life: A longitudinal diffusion tensor imaging study of a South African birth cohort

**DOI:** 10.1016/j.nicl.2024.103572

**Published:** 2024-01-28

**Authors:** K.A. Donald, C.J. Hendrikse, A. Roos, C.J. Wedderburn, S. Subramoney, J.E. Ringshaw, L. Bradford, N. Hoffman, T. Burd, K.L. Narr, R.P. Woods, H.J. Zar, S.H. Joshi, D.J. Stein

**Affiliations:** a Division of Developmental Paediatrics, Department of Paediatrics and Child Health, Red Cross War Memorial Children’s Hospital, University of Cape Town, Cape Town, South Africa; b Neuroscience Institute, University of Cape Town, Cape Town, South Africa; c Department of Psychiatry and Mental Health, University of Cape Town, Cape Town, South Africa; d South African Medical Research Council (SAMRC), Unit on Risk and Resilience in Mental Disorders, University of Cape Town, Cape Town, South Africa; e Department of Paediatrics and Child Health, Red Cross War Memorial Children’s Hospital, University of Cape Town, Cape Town, South Africa; f South African Medical Research Council (SAMRC), Unit on Child and Adolescent Health, University of Cape Town, Cape Town, South Africa; g Ahmanson-Lovelace Brain Mapping Center, Department of Neurology, University of California Los Angeles, Los Angeles, CA, United States; h Department of Psychiatry and Biobehavioral Sciences, University of California Los Angeles, Los Angeles, CA, United States; i The Semel Institute for Neuroscience and Human Behavior, University of California Los Angeles, Los Angeles, CA, United States; j David Geffen School of Medicine, University of California Los Angeles, Los Angeles, CA, United States; k Department of Bioengineering, University of California Los Angeles, Los Angeles, CA, United States

**Keywords:** Early brain development, Prenatal alcohol exposure, White matter maturation, Diffusion tensor imaging

## Abstract

Prenatal alcohol exposure (PAE) can affect brain development in early life, but few studies have investigated the effects of PAE on *trajectories* of white matter tract maturation in young children. Here we used diffusion weighted imaging (DWI) repeated over three time points, to measure the effects of PAE on patterns of white matter microstructural development during the pre-school years. Participants were drawn from the Drakenstein Child Health Study (DCHS), an ongoing birth cohort study conducted in a peri-urban community in the Western Cape, South Africa. A total of 342 scans acquired from 237 children as neonates (N = 82 scans: 30 PAE; 52 controls) and at ages 2–3 (N = 121 scans: 27 PAE; 94 controls) and 6–7 years (N = 139 scans: 45 PAE; 94 controls) were included. Maternal alcohol use during pregnancy and other antenatal covariates were collected from 28 to 32 weeks’ gestation. Linear mixed effects models with restricted maxium likelihood to accommodate missing data were implemented to investigate the effects of PAE on fractional anisotropy (FA) and mean diffusivity (MD) in specific white matter tracts over time, while adjusting for child sex and maternal education. We found significant PAE-by-time effects on trajectories of FA development in the left superior cerebellar peduncle (SCP-L: p = 0.001; survived FDR correction) and right superior longitudinal fasciculus (SLF-R: p = 0.046), suggesting altered white matter development among children with PAE. Compared with controls, children with PAE demonstrated a more rapid change in FA in these tracts from the neonatal period to 2–3 years of age, followed by a more tapered trajectory for the period from 2–3 to 6–7 years of age, with these trajectories differing from unexposed control children. Given their supporting roles in various aspects of neurocognitive functioning (i.e., motor regulation, learning, memory, language), altered patterns of maturation in the SCP and SLF may contribute to a spectrum of physical, social, emotional, and cognitive difficulties often experienced by children with PAE. This study highlights the value of repeated early imaging in longitudinal studies of PAE, and focus for early childhood as a critical window of potential susceptibility as well as an opportunity for early intervention.

## Highlights

• Prenatal alcohol exposure (PAE) alters patterns of white matter tract maturation.
• The superior cerebellar peduncle (SCP) is an important tract affected by PAE in early life.
• Children with PAE had altered rates of FA maturation in the SCP over time, up to age six, compared to unexposed control children.
• Longitudinal scanning time points add value in neuroimaging studies of PAE.

## Graphical abstract

**Figure:**
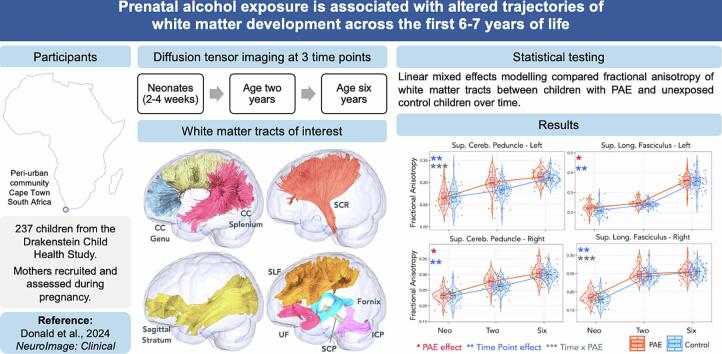

## 1 Introduction

Healthy brain development involves a delicate pattern of complex neurobiological processes that begin early in fetal development and continue throughout childhood (Gilmore et al., 2018). Maternal alcohol use during pregnancy may have teratogenic effects, potentially leading to altered trajectories of child structural and functional neurodevelopment and a wide spectrum of negative neurophysiological outcomes (known as fetal alcohol spectrum disorder [FASD]; Donald et al., 2015, Lebel et al., 2021). Consequently, children with PAE may experience significant long-term difficulties in domains of cognitive, behavioural, and social-emotional functioning (Kar et al., 2021). Investigating the longitudinal effects of PAE on neurodevelopmental trajectories in early childhood may help identify mechanisms that can be targeted for intervention.

Magnetic resonance imaging (MRI) studies, especially those utilizing diffusion weighted imaging (DWI), have significantly broadened our understanding of typical white matter macro- and microstructural development across the lifespan (Ghazi Sherbaf et al., 2019, Lebel and Deoni, 2018). White matter comprises about 50% of brain tissue and consists of billions of nerve cell axons which carry electrical impulses, allowing communication between distant and neighbouring brain regions. The myelin sheaths that surround neuronal axons allow for faster and more efficient transmission of electrical impulses. Although all major white matter tracts begin to develop before birth, myelination occurs predominantly and most rapidly in the first two years of postnatal life and continues at a slower pace up to about 30 years of age (Lebel & Deoni, 2018). DWI metrics—including fractional anisotropy (FA) and mean diffusivity (MD)—are sensitive to the microstructural properties of white matter (e.g., fiber density, myelination).

FA, the most widely reported DWI metric, typically increases with brain development and, until recently, was commonly presumed to reflect increased organization and integrity of white matter tracts, including myelination and fiber density (Gilmore et al., 2018, Lebel et al., 2019, Figley et al., 2022). Following this line of thinking, lower FA was often considered to represent reduced tract microstructural integrity. Recent literature has begun to challenge this assumption, arguing that due to crossing fibers in multiple tracts and the range of factors that may influence the restriction of water diffusion in white matter (i.e., fiber packing density, axon diameter, directional organization, myelination, and membrane permeability; Beaulieu, 2002), caution should be taken when interpreting higher FA as indicating higher microstructural integrity (Figley et al., 2022). This may be especially true in developing populations, where the interpretation of relative value of direction of FA effects may be less clear for the first years of life (Barnea-Goraly et al., 2005). Emerging literature therefore recommends interpreting FA changes alongside more interpretable, non-proportional, and robust DWI metrics such as MD, which quantifies the equally weighted, mean apparent diffusion along all three axes of the diffusion tensor (Figley et al., 2022).

PAE may affect white matter development by altering processes of glial cell proliferation and migration in utero, thereby disrupting the development of myelin sheaths around neuronal axons after birth (Goodlett et al., 2005, Wilhelm and Guizzetti, 2016). Cross-sectional neuroimaging studies have demonstrated the potential effects of PAE on white matter microstructure in association, projection, callosal and brain stem tracts across various age-groups, including infants, children, adolescents, and adults (Ghazi Sherbaf et al., 2019, Kar et al., 2022). One of the most widely reported findings is lower FA of the corpus callosum (CC), the largest commissural tract connecting the left and right hemispheres of the brain (Ghazi Sherbaf et al., 2019). Altered CC microstructure has been associated with poorer cognitive performance, including information processing and eyeblink conditioning in some studies (Fan et al., 2016); however results have been mixed (Lebel et al., 2008). More widespread microstructural changes following PAE have also been reported, specifically of the cerebellar peduncles (Donald et al., 2015, Roos et al., 2021, Fan et al., 2016) and cingulum (Lebel et al., 2008, Paolozza et al., 2017, Santhanam et al., 2011), corticospinal tract, uncinate fasciculus as well as both the inferior and superior longitudinal fasciculi (Donald et al., 2015, Roos et al., 2021).

Due to a lack of longitudinal investigations (Lebel et al., 2021), as well as a paucity of studies in young children specifically (Ghazi Sherbaf et al., 2019, Kar et al., 2022), our understanding of the effects of PAE on early life trajectories of white matter microstructure remains limited. Despite early neuroplasticity, little is known about the extent to which certain white matter microstructural changes detected in infants and very young children with PAE may dissipate or intensify towards middle-childhood. To address these gaps in the literature, we aimed to investigate the effects of PAE on the trajectories of white matter microstructural maturation (using DWI metrics FA and MD) in a cohort of children living in a peri-urban Western Cape community in South Africa, followed from birth to age at school entry (i.e., 6–7 years). In this cohort, which forms part of the Drakenstein Child Health Study (DCHS), we have previously found altered microstructure of the superior longitudinal fasciculus in infants with PAE, with changes in the inferior cerebellar peduncles associated with abnormal neonatal neurobehaviour at the same age (Donald et al., 2015). In a follow-up study at age 2–3 years, we observed microstructural alterations in these same regions, and additionally in the uncinate fasciculus, corticospinal tract, fornix stria terminalis, and sagittal stratum (Roos et al., 2021). Repeated scanning over these early years allows detection of PAE-related changes in white matter microstructure over time, and may elucidate windows of maximal impact of PAE on early trajectories of white matter development.

## 2 Methods

### 2.1 Study design and participants

Participants were part of the Drakenstein Child Health Study (DCHS), an ongoing population-based birth cohort study investigating early determinants of child health and development, conducted in the peri-urban Drakenstein region of the Western Cape, South Africa. The Drakenstein community is comprised of about 200 000 individuals of low socio-economic status living in informal housing, and rates of unemployment are high. More than 90% of women in this region rely on the public health sector for primary health care, including antenatal care and child health services (Stein et al., 2015, Zar et al., 2015). The rate of substance misuse is high in this community, reflecting the broader South African population more generally. Indeed, South Africa has the highest reported prevalence of FASD in the world, with rates as high as 310 per 1000 children living in rural communities (May et al., 2022).

The procedures of the DCHS have been described in detail elsewhere (Stein et al., 2015, Zar et al., 2015). Briefly, pregnant women aged 18 years or older were recruited at 20–28 weeks’ gestation while attending routine antenatal care at one of two primary healthcare clinics in the Drakenstein area. Mothers provided written informed consent for themselves and their children to participate in the study, and background data were collected (i.e., socio-demographics, psychosocial risk factors). Ethics approval for the main DCHS and this brain imaging sub-study were obtained from the University of Cape Town Health Sciences Faculty, Human Research Ethics Committee (401/2009, 525/2012, 044/2017) and the Western Cape Department of Provincial Health Research Committee (2011RP45).

**Fig. 1:**
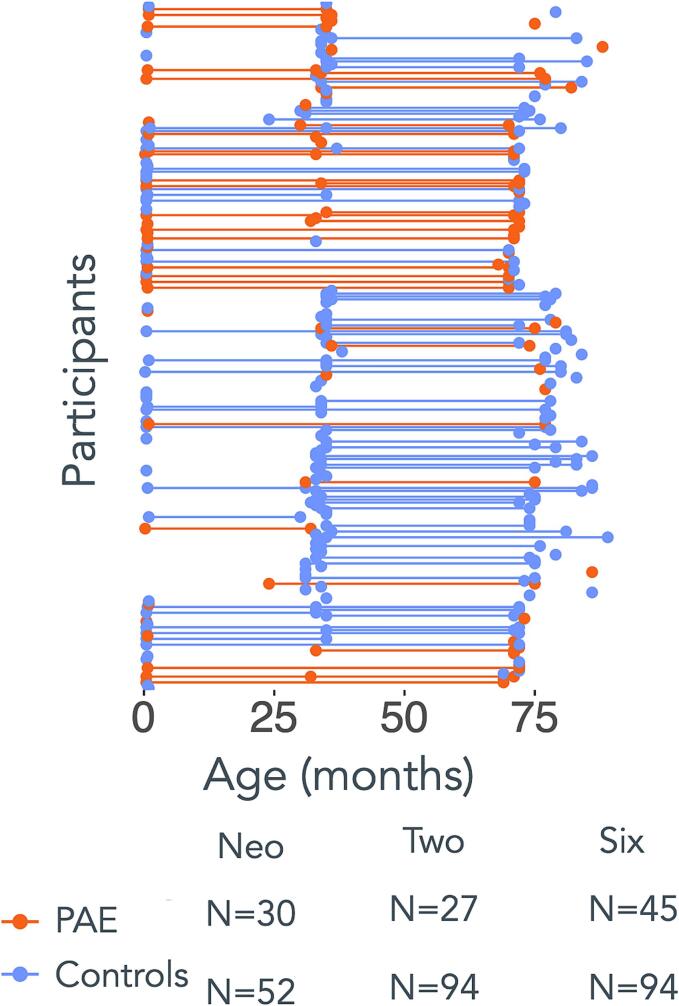
Age and time between scans by prenatal alcohol exposure (PAE) status. Each dot represents a scan (N = 342), connecting lines represent the time between scans, and colour indicates the PAE status for each participant.

A subset of children with and without PAE from mothers participating in the DCHS were included in the current brain imaging sub-study and scanned at three time points over the first 6–7 years of life. Exclusion criteria for the children were: (i) the mother had a positive urine toxicology result during pregnancy for drugs of abuse besides alcohol and tobacco (i.e., illicit substances including cannabis, cocaine, methamphetamines, methaqualone, opiates, and MDMA), (ii) low Apgar score (<7 at 5 min), (iii) admittance to the neonatal intensive care unit, (iv) an identified genetic syndrome or congenital abnormalities, (v) HIV infection, and/or (vi) standard MRI contraindications (Donald et al., 2015, Wedderburn et al., 2020). Supplementary Fig. 1 presents a breakdown of participant dropout and exclusion rates for the three time points. A total of 342 scans acquired on 237 children (67 PAE, 170 controls) across the three time points were included in the current analysis comprising 82 neonates (30 PAE, 52 controls), 121 2–3-year-olds (27 PAE, 94 controls), and 139 6–7-year-olds (45 PAE, 94 controls). Fig. 1 depicts the age and time between scans for all included children by PAE status. Fifty-nine percent of the children (N = 140: 34 PAE, 106 controls) were scanned at one time point, while 41 percent (N = 97: 33 PAE, 64 controls) were scanned at two or three time points (Fig. 1).

### 2.2 Maternal assessments

All mothers completed the World Health Organization’s Alcohol, Smoking and Substance Involvement Screening Test (ASSIST) between 28 and 32 weeks’ gestation to assess comorbid substance use (Humeniuk et al., 2008). This self-report questionnaire assesses a range of substances (e.g., alcohol, tobacco, cannabis, cocaine, amphetamine-type stimulants, inhalants, sedatives/sleeping pills, hallucinogens, opioids, and others) and has been validated for use in South Africa (Jackson et al., 2010). The children born to mothers with a total ASSIST score above 11 on the alcohol questions, and/or who indicated moderate-severe alcohol use during pregnancy (i.e., >2 drinks twice per week in any trimester), were categorized as alcohol exposed (i.e., PAE group). Data on the frequency and quantity of maternal alcohol use during each pregnancy trimester was collected at the neonatal imaging visit and is reported in Table 1. Maternal urine cotinine levels were additionally measured to determine active tobacco smoking status during pregnancy (i.e., cotinine level > 500 ng/mL). Mothers also completed the Beck Depression Inventory during pregnancy, and the Edinburgh Postnatal Depression Scale at six months postpartum, to measure prenatal and postnatal depressive symptomatology (Beck et al., 1988, Cox et al., 1987, Tsai et al., 2013).

**Table 1:** Frequency and quantity of maternal alcohol use during each pregnancy trimester for the three time points.

	T1: Neonates (PAE: n = 30)			T2: 2–3-year-olds (PAE: n = 20)^a^			T3: 6–7-year-olds (PAE: n = 36)^a^		
	**Trim. 1**	**Trim. 2**	**Trim. 3**	**Trim. 1**	**Trim. 2**	**Trim. 3**	**Trim. 1**	**Trim. 2**	**Trim. 3**
Alcohol usage, n (%) Once per week or less 2 to 3 times per week	22 (73) 19 3	12 (40) 9 3	7 (23) 4 3	16 (80) 14 2	9 (45) 7 2	5 (25) 3 2	27 (75) 21 6	17 (47) 12 5	11 (31) 6 5
Number of drinks per occasion <2 2 to 3 4 or more	1 6 15	1 4 7	0 2 5	0 5 11	0 5 4	0 2 3	0 10 17	1 7 9	1 1 9

Abbreviations: T1, T2, T3, time point 1, 2, 3; PAE, prenatal alcohol exposure; Trim., trimester.

a Data on the timing, frequency, and quantity of maternal drinking during pregnancy was collected only at the neonatal imaging visit. Therefore, this data is missing for participants who did not attend neonatal imaging, but who attended imaging at the second and third time points. There was missing data for 7 participants at the 2–3-year time point, and 9 participants at the 6–7-year time point.

### 2.3 Child brain imaging

Imaging protocols were tailored for each of the three time points to be optimal for the children’s developmental stage (Roos et al., 2022). Table 2 describes the scanner specifications, imaging modalities, and acquisition parameters relevant to each time point. Two DWI sets were obtained at each time point, in the transverse plane with both anterior–posterior and posterior–anterior phase encoding to control for anatomic distortions and increase signal-to-noise. T2-weighted (time point 1) or T1-weighted (time points 2 and 3) structural MRI (sMRI) scans were also collected and provided an individual anatomical reference volume for each child during image processing. Anthropometric data (i.e., weight, height, head circumference) were collected at each time point, and health information at birth were extracted from hospital records. Children were asleep during scanning at the first two time points, but awake and quiet during the last.

**Table 2:** Imaging protocol for each time point.

**Time point**	**Neonates**	**2**–**3-year-olds**	**6**–**7-year-olds**
**MRI scanner**	3 T Siemens Allegra at CUBIC-Tygerberg	3 T Siemens Skyra at CUBIC-UCT	3 T Siemens Skyra at CUBIC-UCT
**Head coil**	Single-channel	32-channel	32-channel
**Diffusion Weighted Imaging** Gradient directions Repetition time Echo time Voxel size Slice thickness B-values Field of view Scan duration	45 7900 ms 90 ms 1.3x1.3x1.6 mm^3^ 1.6 mm 0 and 1000 s/mm^2^ 160x160x97mm 2 x 6min27s	30 7800 ms 92 ms 1.8x1.8x2mm^3^ 2 mm 0 and 1000 s/mm^2^ 230x230x121mm 2 x 8min36s	30 9800 ms 92 ms 2x2x2mm^3^ 2 mm 0 and 1000 s/mm^2^ 240x240x122mm 2 x 6min41s
**Structural MRI** Sequence Weighting Repetition time Echo time Flip angle Voxel size Field of view Slices Slice thickness Scan duration	tse3d_sag T2 3500 ms 354 ms 180 degrees 1x1x1mm^3^ 160x160x128mm 128 1 mm 5min41s	MEMPRAGE T1 2530 ms 1.69, 3.54, 5.39, 7.24 ms 7 degrees 1x1x1mm^3^ 224x224x176mm 176 1 mm 5min21s	MEMPRAGE T1 2500 ms 3.35 ms 8 degrees 1x1x1mm^3^ 256x256x176mm 176 1 mm 7min12s

Abbreviations: CUBIC, Cape Universities Brain Imaging Centre; UCT, University of Cape Town.

### 2.4 Image processing

All MRI data were quality checked before preprocessing. Participants had to have at least one structural MRI scan and one DWI scan in each phase-encoding direction without disruptive movement or other artifacts for inclusion. DWI scans were preprocessed on the Centre for High Performance Computing cluster (CHPC, Cape Town) using TORTOISE software (Irfanoglu et al., 2018, Pierpaoli et al., 2010), which was chosen due to its superior correction and registration ability in pediatric samples prone to movement during scanning (Roos et al., 2021, Taylor et al., 2016). In the neonates, T2-weighted sMRI images were registered to the University of North Carolina (UNC) neonate structural template (Shi et al., 2011) given the relatively immature state of tract formation compared to the other time points (Roos et al., 2022). The registered image was then used as an anatomical reference image in TORTOISE. In the other time points, T1-weighted sMRI images were inverted to have similar contrast to the diffusion b0 volume, and used as an anatomical reference volume in TORTOISE. The DIFF PREP and DR BUDDI modules of TORTOISE were used, respectively, to (i) compute motion, eddy currents, and echo-planar imaging (EPI) distortion corrections, and (ii) merge encoded sets and perform further EPI distortion corrections.

After preprocessing, the Tract-based Spatial Statistics (TBSS) pipeline of FMRIB Software Library (FSL) was used to perform diffusion tensor parameter fitting and to extract diffusion parameters (Smith et al., 2006). Preprocessed FA images were first created by applying brain extraction using BET and tensor extraction using DTIFIT (Smith, 2002). Thereafter, the 4 steps of TBSS were applied as described in Roos et al. (2021). Importantly, since we had a pediatric sample for whom the use of an adult MNI template would be inappropriate, we used study-specific templates as the registration target in the second step of TBSS within each time point. The use of a study-specific template significantly enhances registration quality (Bach et al., 2014, Smith et al., 2006). Finally, the mean FA skeleton was used to map and derive MD images, and summary metrics were extracted for 48 white matter tracts using the Johns Hopkins University ICBM-DTI-81 atlas (Mori et al., 2008).

### 2.5 Statistical analysis

**Fig. 2:**
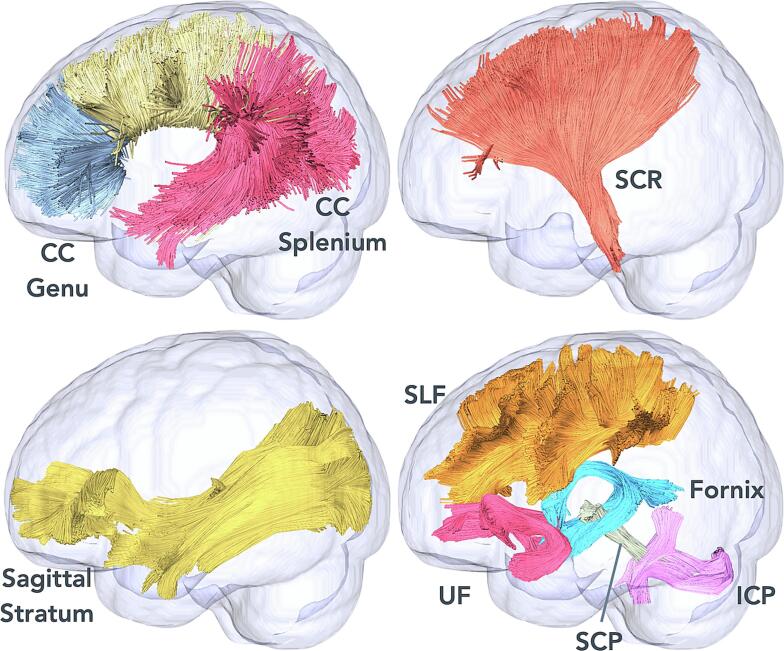
Illustration of investigated tracts on glass brains. CC = corpus callosum, SCR = superior corona radiata, SLF = superior longitudinal fasciculus, UF = uncinate fasciculus, SCP = superior cerebellar peduncle, ICP = inferior cerebellar peduncle.

We conducted all statistical analyses in R version 4.2.1. Linear mixed effects models were fitted with restricted maxium likelihood (REML) to accommodate missing data, and implemented using lme4 (Bates et al., 2015) and lmerTest (Kuznetsova et al., 2017). We tested the effects of PAE, time, and the interaction of PAE with time as predictors while controlling for sex and maternal education as regressors on FA and MD of those salient white matter tracts implicated by PAE in our prior work. Fig. 2 illustrates the investigated tracts, which included the left and right superior longitudinal (SLF-L/R) and uncinate fasciculus (UF-L/R), left and right sagittal stratum (SS-L/R), left and right inferior (ICP-L/R) and superior cerebellar peduncles (SCP-L/R), corpus callosum genu (CC-G) and splenium (CC-S), fornix, and left and right superior corona radiata (SCR-L/R). The participant was included as a random effect. Specifically, the fixed effects model was given by FA ∼ PAE status + Time + Sex + Maternal education + (PAE status x Time). We tested the omnibus model with all three time points together followed by pairwise comparisons between two time points, separately for neonates and 2–3-year-old children, neonates and 6–7-year-old children, and 2–3-year-old and 6–7-year-old children. Post-hoc cross-sectional analysis for group differences between children with PAE and controls within each time point was performed with ANOVA after adjusting for sex and maternal education. Additionally, post-hoc sensitivity analyses were performed to test the robustness of our findings while including prenatal tobacco exposure as an additional covariate, given its known association with white matter microstructure in this cohort (Roos et al., 2021). We used the Benjamini Hochberg false discovery rate (FDR) to correct for multiple comparisons within each diffusion measure across all tracts, though we report findings that survived correction and those that did not, given the strong a priori inclusion of these tracts based on findings in our own cohort.

## 3 Results

### 3.1 Socio-demographic and psychosocial group differences

Table 3 outlines the socio-demographic and psychosocial characteristics of PAE and control groups. PAE and control groups were similar in terms of sex and gestational age at delivery within each time point. A 4-month difference in mean age was present at the 6–7-year time point, but not at the neonatal and 2–3 year points, with PAE children being slightly younger than controls at scanning. However, the age range of the PAE and control groups were similar (68–88 and 69–89 months respectively). Similarly, children with PAE had smaller anthropometric measurements in terms of height, weight, and head circumference than controls at the 6–7 year time point only. Maternal tobacco use during pregnancy was higher in PAE children compared to controls for all three time points, however we tested the influence of PTE on our main findings in a post-hoc sensitivity analysis. Maternal characteristics were generally matched for children with PAE and controls, in terms of age at enrolment (except for time point 3), educational attainment, employment, monthly income, marital status, household size, unplanned pregnancy, and pre- and postnatal depression. Even though our sample included children who were prenatally exposed to maternal HIV infection and maternal antiretroviral therapy (ART) use during gestation, none of the children in the final sample were HIV infected as confirmed through testing after birth, and HIV/ART-exposure was balanced across groups for all three time points.

**Table 3:** Socio-demographic characteristics and psychosocial risk factors of children and mothers across the three time points.

Variable^a^, ^b^	T1: Neonates (N = 82)			T2: 2–3-year-olds (N = 121)			T3: 6–7-year-olds (N = 139)		
	**PAE** **(n = 30)**	**Control (n = 52)**	***p***	**PAE** **(n = 27)**	**Control (n = 94)**	***p***	**PAE** **(n = 45)**	**Control (n = 94)**	***p***
Child socio-demographics									
Age at scanning: T1 in weeks, T2 and T3 in months	3 ± 1 (1–4)	3 ± 1 (1–5)	0.170	34 ± 2 (30–36)	34 ± 2 (30–38)	0.338	73 ± 4 (68–88)	77 ± 5 (69–89)	<0.001
Sex, boys	14 (47)	33 (64)	0.139	15 (56)	57 (61)	0.635	22 (49)	52 (55)	0.477
Gestation in weeks	39 ± 2 (34–42)	39 ± 2 (35–41)	0.300	39 ± 2 (34–42)	39 ± 3 (28–42)	0.366	38 ± 3 (30–42)	39 ± 2 (31–42)	0.100
Child anthropometry									
Height in centimetres^c^	51 ± 5 (45–65)	49 ± 3 (42–56)	0.032	92 ± 4 (88–102)	92 ± 4 (85–100)	0.833	113 ± 5 (100–121)	118 ± 6 (105–128)	<0.001
Weight in kilograms^d^	4 ± 1 (3–5)	4 ± 1 (3–5)	0.724	13 + 2 (11–18)	14 ± 2 (11–20)	0.099	19 ± 2 (14–24)	22 ± 4 (17–33)	<0.001
Head circumference in centimetres^e^	36 ± 1 (34–38)	37 ± 2 (31–40)	0.092	49 ± 2 (46–52)	50 ± 2 (47–55)	0.029	51 ± 2 (48–55)	52 ± 2 (48–56)	<0.001
Maternal socio-demographics at enrolment									
Age in years	26 ± 6 (18–39)	26 ± 6 (18–39)	0.787	27 ± 6 (18–41)	27 ± 6 (18–45)	0.860	26 ± 6 (18–39)	28 ± 6 (18–45)	0.022
Educational attainment Primary Some secondary Completed secondary Any tertiary	1 (3)18 (60)10 (33)1 (3)	4 (8)22 (42)24 (46)2 (4)	0.465	4 (15)17 (63)6 (22)0 (0)	2 (2)58 (62)29 (31)5 (5)	0.051	5 (11)25 (56)13 (29)2 (4)	3 (3)53 (56)36 (38)2 (2)	0.178
Employed	11 (37)	17 (33)	0.715	6 (22)	26 (28)	0.572	14 (31)	31 (33)	0.826
Monthly income <ZAR1000 ZAR1000-ZAR5000 >ZAR5000	23 (77)7 (23)0 (0)	35 (67)16 (31)1 (2)	0.753	23 (85)4 (15)0 (0)	74 (79)20 (21)0 (0)	0.589	38 (84)7 (16)0 (0)	75 (80)16 (17)3 (3)	0.699
Married/cohabitating	9 (30)	23 (44)	0.203	13 (48)	41 (44)	0.676	14 (31)	41 (44)	0.158
Household size	5 ± 2 (3–12)	5 ± 2 (1–11)	0.296	5 ± 2 (1–11)	4 ± 2 (1–14)	0.143	5 ± 2 (1–12)	5 ± 3 (1–13)	0.122
Maternal psychosocial risk factors									
Unplanned pregnancy^f^	23 (77)	37 (76)	0.907	16 (62)	49 (63)	0.907	30 (70)	48 (62)	0.365
Tobacco use during pregnancy	19 (63)	15 (29)	0.002	20 (74)	20 (21)	<0.001	30 (67)	20 (21)	<0.001
HIV infection	6 (20)	13 (25)	0.605	7 (26)	41 (44)	0.098	11 (24)	37 (39)	0.084
Antenatal depression (BDI): above threshold^f^	9 (30)	13 (27)	0.738	7 (27)	16 (21)	0.495	11 (26)	18 (23)	0.757
Postnatal depression six months postpartum (EPDS): above threshold^g^	7 (32)	7 (24)	0.543	3 (19)	6 (12)	0.430	7 (22)	10 (18)	0.646

Abbreviations: T1, T2, T3, time point 1, 2, 3; PAE, prenatal alcohol exposure; ZAR, South African rand; HIV, human immunodeficiency virus; BDI, Beck Depression Inventory; EPDS, Edinburgh Postnatal Depression Scale.

a Values for continuous variables are presented as: mean ± standard deviation (range). Values for categorical variables are presented as: number (%).

b Statistical differences between children with PAE and controls were calculated within each time point using independent samples *t* tests for continuous variables and Pearson chi-square tests or Fischer’s exact tests (as appropriate) for categorical variables (significance at *p* < 0.05, not corrected for multiple comparisons).

c Missing height data: T1 (n = 2), T2 (n = 17), T3 (n = 1).

d Missing weight data: T1 (n = 2), T2 (n = 1).

e Missing head circumference data: T1 (n = 2), T2 (n = 1).

f Missing pregnancy-related and BDI data: T1 (n = 3), T2 (n = 17), T3 (n = 18).

g Missing EPDS data: T1 (n = 31), T2 (n = 53), T3 (n = 51).

### 3.2 PAE effects on FA and MD across development

**Fig. 3:**
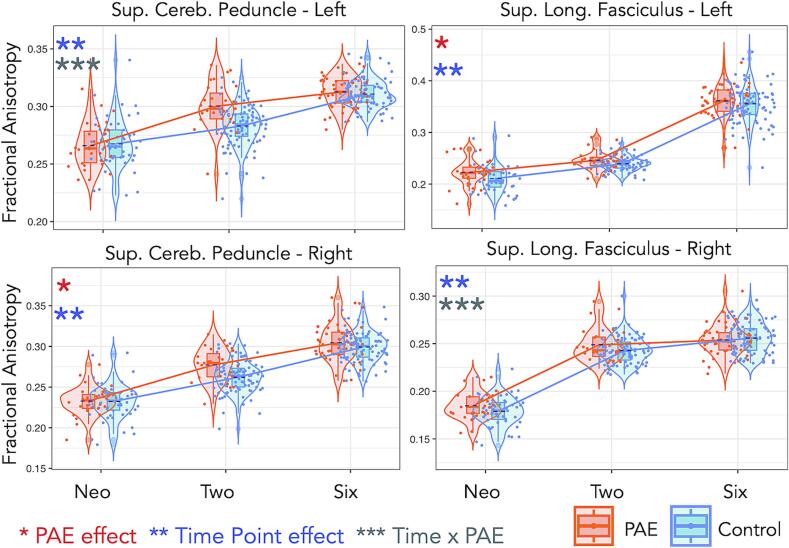
Trajectories of fractional anisotropy (FA) for the left and right tracts of the superior cerebellar peduncle and the superior longitudinal fasciculus for both PAE and controls through birth, 2–3-, and 6–7-year time points in development. The SCP-L was significant after correction. One star (*) shows a significant PAE effect, two stars (**) show a time effect, and three stars (***) show a PAE-by-time interaction effect. Jitter is added for visualization purposes (only in the time point direction).

**Fig. 4:**
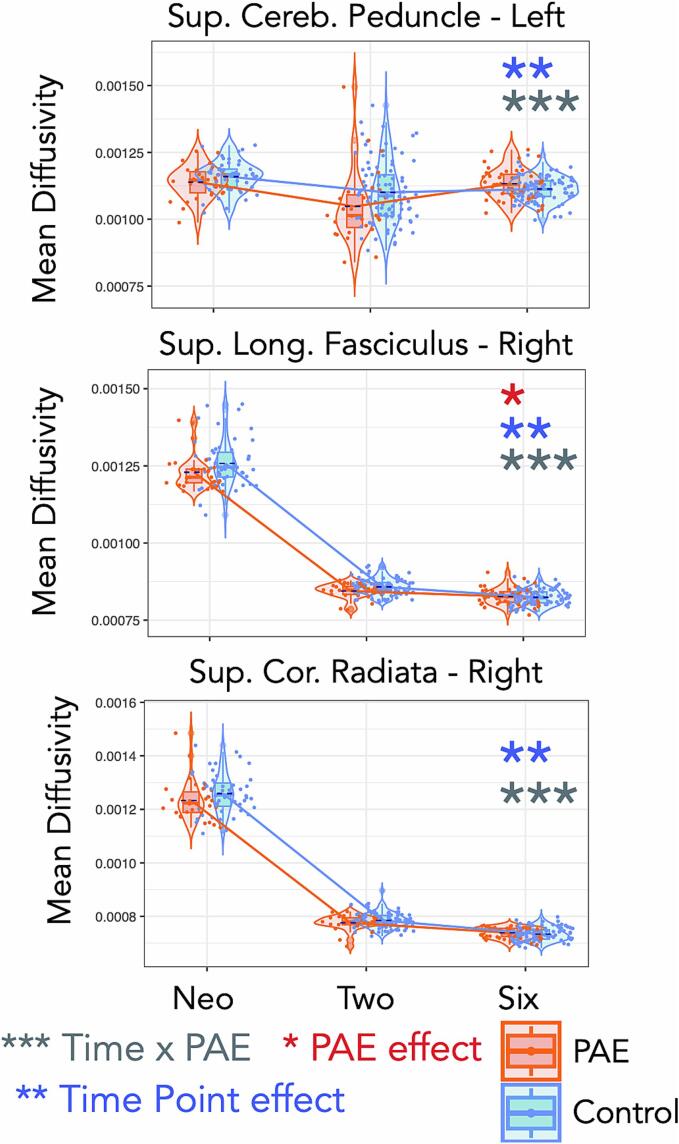
Trajectories of mean diffusivity (MD) for the left superior cerebellar peduncle, and the right superior longitudinal fasciculus and superior corona radiata for both PAE and controls through birth, 2–3-, and 6–7-year time points in development. One star (*) shows a significant PAE effect, two stars (**) show a time effect, and three stars (***) show a PAE-by-time interaction effect (not corrected for multiple comparisons). Jitter is added for visualization purposes (only in the time point direction).

We found significant main effects of PAE on FA and MD across the period from birth to 6–7 years (see Table 4 for a summary of results or Supplementary Table 1 for full results). Children with PAE demonstrated greater FA in the SCP-L/R (p = 0.021; Fig. 3), ICP-R (p = 0.011), SS-L (p = 0.001), and SLF-L (p = 0.025; Fig. 3), and reduced MD in the SLF-R (p = 0.031; Fig. 4), compared with controls, though these findings did not survive FDR corection. Starting with the neonates, the differences in FA between children with PAE and controls were minimal; however, FA in the 2–3-year-old children with PAE was higher than that of the controls. These differences decreased again as both the groups reached 6–7 years of age.

**Table 4:** Longitudinal effects of PAE, Time, and PAE-by-time interaction on FA and MD.

**Tract**	**L/R**	**Fractional Anisotropy (FA)^a^**						**Mean diffusivity (MD)^a^**					
		**PAE**		**Time**		**PAE x Time**		**PAE**		**Time**		**PAE x Time**	
		**Partial eta^2^**	***p***	**Partial eta^2^**	***p***	**Partial eta^2^**	***p***	**Partial eta^2^**	***p***	**Partial eta^2^**	***p***	**Partial eta^2^**	***p***
Superior longitudinal fasciculus	L	0.016	0.025	0.842	<0.001*	–	ns	–	ns	0.962	<0.001*	–	ns
	R	–	ns	0.864	<0.001*	0.027	0.046	0.016	0.031	0.951	<0.001*	0.022	0.038
Sagittal stratum	L	0.039	0.001	0.881	<0.001*	–	ns	–	ns	0.928	<0.001*	–	ns
	R	–	ns	0.897	<0.001*	–	ns	–	ns	0.957	<0.001*	–	ns
Uncinate fasciculus	L	–	ns	0.923	<0.001*	–	ns	–	ns	0.935	<0.001*	–	ns
	R	–	ns	0.820	<0.001*	–	ns	–	ns	0.690	<0.001*	–	ns
Inferior cerebellar peduncle	L	–	ns	0.563	<0.001*	–	ns	–	ns	0.744	<0.001*	–	ns
	R	0.026	0.011	0.512	<0.001*	–	ns	–	ns	0.262	<0.001*	–	ns
Superior cerebellar peduncle	L	0.023	0.007	0.522	<0.001*	0.042	0.001*	–	ns	0.109	<0.001*	0.035	0.008
	R	0.025	0.021	0.784	<0.001*	–	ns	–	ns	0.477	<0.001*	–	ns
Corpus callosum genu	n/a	–	ns	0.954	<0.001*	–	ns	–	ns	0.951	<0.001*	–	ns
Corpus callosum splenium	n/a	–	ns	0.924	<0.001*	–	ns	–	ns	0.978	<0.001*	–	ns
Fornix	n/a	–	ns	0.248	<0.001*	–	ns	–	ns	0.037	0.039	–	ns
Superior corona radiata	L	–	ns	0.926	<0.001*	–	ns	–	ns	0.968	<0.001*	–	ns
	R	–	ns	0.924	<0.001*	–	ns	–	ns	0.965	<0.001*	0.021	0.047

Abbreviations: PAE, prenatal alcohol exposure; L/R, left or right hemisphere; n/a, not applicable; ns, not significant at p < 0.05.

**^a^**Model adjusting for sex and maternal education.

* Survived false discovery rate (FDR) correction for multiple comparison.

### 3.3 Time effects on FA and MD across development

As expected, all tracts showed a significant main effect of time (p < 0.0001, survived multiple comparisons) for both FA and MD throughout development from the neonatal age to 6–7 years (Table 4, Supplementary Table 1). FA increased with age, while MD decreased with age.

### 3.4 Pae-by-time effects on FA and MD across development

We found a significant PAE-by-time interaction effect for FA in the SCP-L (p = 0.001) that survived correction for multiple comparisons, after adjusting for sex and maternal educational attainment. This differential effect in the rate of change of white matter maturation shows a more rapid increase of FA in children with PAE from the neonatal period to 2–3 years of age, and then a more tapered change for the period from 2–3 to 6–7 years of age compared to controls. Conversely, the rate of change in FA was slower in controls in the initial neonatal period up to 2–3 years, and slightly higher from 2–3 to 6–7 years. We also found a PAE-by-time interaction effect for FA in the SLF-R (p = 0.046), which did not survive FDR correction. For this tract, FA showed a minimal rate of change for both groups from 2–3 to 6–7 years of age. Fig. 3 shows trajectories of FA development across the three time points for the SCP-L/R and SLF-L/R.

We found PAE-by-time interaction effects for MD in the SLF-R (p = 0.038), SCP-L (p = 0.008), and SCR-R (p = 0.047), after adjusting for sex and maternal educational attainment, however, these effects did not survive multiple comparison correction. Fig. 4 shows trajectories of MD development across the three time points for these tracts. Complementary to the effects on FA, we observed that MD was lower in children with PAE compared with controls across all time points (except the SCP-L at age 6–7 years) in these tracts.

### 3.5 Post-hoc pairwise comparisons of time points

In a post-hoc analysis, we compared pairwise time points for neonates and 2–3 years, neonates and 6–7 years, and 2–3 and 6–7 years using the same interaction model as defined previously.

#### 3.5.1 Neonates and 2–3-year-olds

We found significant interaction effects between PAE and time for FA in the SCP-L (p = 0.004), SCP-R (p = 0.039), and UF-R (p = 0.032). We also found significant effects of PAE across the neonatal period to 2–3 years for FA in the SCP-L/R (p = 0.019, p = 0.026), SS-L/R (p = 0.011, p = 0.037), and SLF-L/R (p = 0.007, p = 0.015) and for MD in the SCP-L (p = 0.039), and SLF-R (p = 0.027). These effects did not survive FDR correction (Supplementary Table 2).

#### 3.5.2 Neonates and 6–7-year-olds

There were no significant interaction effects between PAE and time on FA. Significant PAE-by-time effects on MD were evident in the SLF-R (p = 0.028), SCP-L (p = 0.010), and SCR-R (p = 0.033). Moreover, we found nominally significant effects (uncorrected) of PAE for FA in the ICP-R (p = 0.033) and SS-L (p = 0.021). None of these effects survived multiple comparisons corrections (Supplementary Table 3).

#### 3.5.3 2–3-year-olds and 6–7-year-olds

**Fig. 5:**
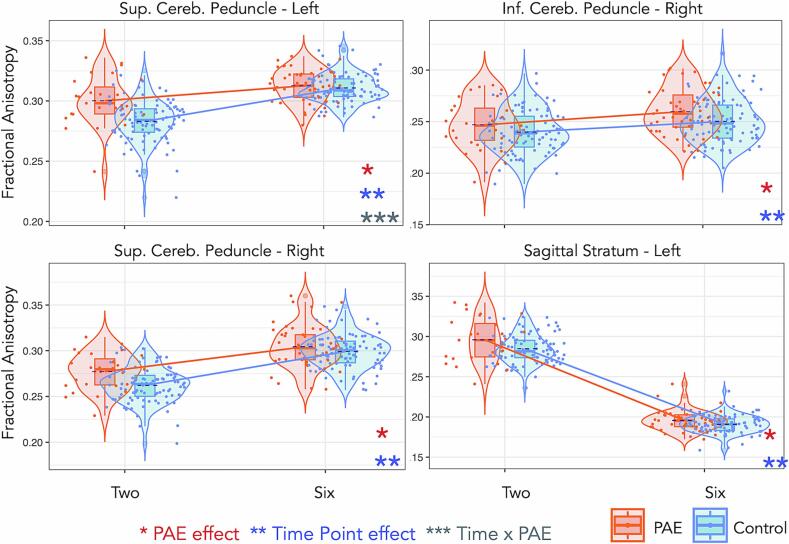
Trajectories of fractional anisotropy (FA) for the left and right tracts of the superior cerebellar peduncle, the left inferior cerebellar peduncle, and the left sagittal stratum for both PAE and unexposed controls through 2–3 and 6–7 years. One star (*) shows a significant PAE effect, two stars (**) show a time effect, and three stars (***) show a PAE-by-time interaction effect. All effects were significant after multiple comparisons correction.

We found an interaction effect between PAE and time for FA in the SCP-L (p = 0.0007), which was significant after multiple comparisons correction, as shown in Fig. 5. The interaction effects for FA in the CC-G (p = 0.048), SCR-L (p = 0.017), SLF-R (p = 0.008), and fornix (p = 0.022) did not survive correction. Significant PAE effects (corrected for multiple comparisons) were found for FA in the SCP-L/R (p < 0.001, p = 0.003), ICP-R (p = 0.010), and SS-L (p = 0.0008) (Fig. 5). We also found significant PAE-by-time effects on MD in the SCP-L (p = 0.005), SS-R (p = 0.010), and SLF-L (p = 0.001), which survived multiple comparisons correction, whereas PAE-by-time effects on MD in the SLF-R (p = 0.028), CC-G (p = 0.030), and SCR-L (p = 0.042) did not survive correction (Supplementary Table 4).

### 3.6 Cross-sectional PAE effects on FA and MD within each time point

Table 5 provides a summary of PAE effects on FA and MD within each time point. At the neonatal age, no significant differences were found in FA or MD in any of the tracts. At the 2–3-year time point, children with PAE demonstrated significantly greater FA than controls in the SCP-L/R (p = 0.00015, p = 0.0015) when corrected for multiple comparisons. They also had increased FA in the SS-L/R (p = 0.017, p = 0.048), and SLF-L/R (p = 0.038, p = 0.041) and decreased MD in the SS-L (p = 0.015) and SLF-L/R (p = 0.014, p = 0.048) compared with controls, however, these differences failed to achieve significance after correction. Lastly, at 6–7 years of age, children with PAE showed higher FA in the ICP-R (p = 0.020) and SS-L (p = 0.019), and higher MD in the SCP-L (p = 0.045) compared with controls, which were not significant after correction for multiple comparisons.

**Table 5:** Summary of cross-sectional PAE effects on FA and MD within each time point.

		**Neonates^a^** **(N = 82)**		**2**–**3-year-olds^a^** **(N = 121)**		**6**–**7-year-olds^a^** **(N = 139)**	
		**PAE effect**	***p***	**PAE effect**	***p***	**PAE effect**	***p***
Superior longitudinal fasciculus	L	–	ns	↑ FA ↓ MD	0.038 0.014	–	ns
	R	–	ns	↑ FA ↓ MD	0.041 0.048	–	ns
Sagittal stratum	L	–	ns	↑ FA ↓ MD	0.017 0.015	↑ FA	0.019
	R	–	ns	↑ FA	0.048	–	ns
Inferior cerebellar peduncle	R	–	ns	–	ns	↑ FA	0.0197
Superior cerebellar peduncle	L	–	ns	↑ FA	0.00015*	↑ MD	0.045
	R	–	ns	↑ FA	0.00153*	–	ns

Abbreviations: PAE, prenatal alcohol exposure; FA, fractional anisotropy; MD, mean diffusivity; L/R, left or right hemisphere; ns, not significant.

^a^Model adjusting for sex and maternal education.

* Survived FDR correction for multiple comparisons.

### 3.7 Post-hoc sensitivity analysis with prenatal tobacco exposure

We tested the robustness of our longitudinal trajectory findings for FA in the SCP and SLF by additionally adjusting for prenatal tobacco exposure (PTE). A chi-square test comparing nested models that included PTE, retained the significance of the PAE-by-time effect for FA in the SCP-L (p = 0.002), corrected for multiple comparisons. The (uncorrected) PAE-by-time effect in the SLF-R (p = 0.042) was also retained (Supplementary Table 5).

## 4 Discussion

Our data suggest that effects of PAE influence white matter maturational processes throughout early childhood, a critical window for neurodevelopment. Both the PAE and control groups demonstrated significant increases in FA and decreases in MD in all tracts from the neonatal period to age 6–7 years, consistent with previous longitudinal studies of DTI during this period (Treit et al., 2013). However, children with PAE showed differential rates of FA and MD change in specific tracts, including the SCP-L and SLF-R, compared with controls over the study period. Children with PAE demonstrated generally higher FA in these tracts at age 2–3 years. These changes, measured across three time points in children in a birth cohort study in South Africa, suggest that PAE impacts white matter microstructure persistently across the first 6–7 years of life. This is the first study that, being of sufficient duration, has reported an association between PAE and white matter from birth to school age in the same cohort.

Incorporating early-life imaging of children into a longitudinal study has allowed us to identify specific time points when maximum PAE-related white matter changes are detectable in the early years. As evidenced by significant PAE-by-time effects over the three time points, this study has found that in specific regions there was a difference in the trajectory of change in white matter maturation over the pre-school years. Both the SCP-L and the SLF-R demonstrated altered trajectory patterns in FA and MD for children with PAE compared to controls. Imaging data captured at critical time points over this 6–7-year period allowed us to observe relative change across early childhood through post-hoc pairwise comparisons.

On examination of pairwise time points, when including only the neonatal time point and the 6–7-year time point (without including the middle, 2–3-year time point), none of the tracts in our model demonstrated a difference in the FA or MD trajectory. Cross-sectional differences at the neonatal and 6–7-year time points were minimal compared to the 2–3-year time point–children with PAE showed greater FA in the SS-L and ICP-R at age 6–7 years, as well as increased MD in the SCP-L at age 6–7 years. However, none of these associations survived multiple comparisons correction.

When looking at the neonatal to 2–3-year time points, children with PAE demonstrated altered FA and/or MD maturation in the SLF-L/R, SS-L/R, and SCP-L/R, resulting in cross-sectional group differences for FA in all of these tracts at age 2–3 years. The maturation of white matter is most rapid during the first two years of postnatal life as reflected in the dramatic changes in metrics describing white matter microstructural change over this time (Gilmore et al., 2018, Girault et al., 2019). It is unsurprising therefore, that during this period of high-intensity change, diffusion imaging would be sensitive to a wide group of regions potentially impacted by PAE.

Likewise, when only the 2–3- and 6–7-year time points were included, children with PAE showed differential rates of FA and/or MD change in the SLF-L/R, SS-R, SCP-L (FA change survived multiple comparisons correction). Group differences in all of these tracts were evident at the 2–3-year time point, but only in the SCP-L at the 6–7-year time point. As such, we observed a tapering of group differences that occurred from birth to age 2–3 years during the developmental period of 2–3 to 6–7 years. In general, for the concerned tracts, children with PAE demonstrated a slower rate of FA maturation than controls from the second to third time points, while FA growth in the controls continued to increase at a more rapid pace. These altered patterns of FA maturation between children with PAE and controls across the first 6–7 years of life would not have been observed without intermediate imaging time points. As such, our findings highlight the value of studies investigating not only measures across early life time periods, but repeating through childhood and adolescence in order to better understand the effects of PAE on longitudinal patterns of brain growth and maturation.

Key regions which appear to have the most robust and persistent trajectory changes across this period were the SLF and SCP bilaterally. These regions have been well-described as being affected by PAE (Fan et al., 2016, Lebel et al., 2008, Paolozza et al., 2017, Treit et al., 2013) and play important roles in neural networks serving language function, visuospatial functioning, motor regulation, learning, and memory(Lebel et al., 2012, Mattson et al., 2001). While this study is consistent with previous research in finding PAE trajectory effects in these important regions, it has also found a more marked rate of change in white matter development between PAE and unexposed children in early life (0–2–3 years), compared to the later pre-school years (2–3 to 6–7 years).

The SLF, present in each cerebral hemisphere, is one of the major white matter association tracts connecting parietal, occipital and temporal lobes with ipsilateral frontal cortices. It is known to play a role in networks serving language function, visuospatial functioning and working memory and to be critical for integrating information from multiple sensory modalities essential for complex cognitive processes. Lebel and colleagues (2008) have demonstrated that PAE is associated with altered development of the SLF during childhood and adolescence, with decreased FA in some segments of the SLF and increased FA in others. Our findings extend these previous studies into infancy, showing that the effects of PAE on SLF white matter maturation are seen by 2 years of age, are particularly impactful on the maturation trajectory during this early period, and have ongoing impact throughout childhood.

The SCP conveys selective afferents to the cerebellum and carries extensive efferent connections from all the cerebellar nuclei to the thalamus and other deep grey nuclei, serving important pathways for motor regulation, learning, and memory(Lebel et al., 2012, Mattson et al., 2001). The SCP is also involved in several cognitive functions, including attention, visuospatial abilities, and working memory. Previous research has suggested that PAE can affect the development of the SCP during early life, with alterations in microstructural properties persisting into adolescence (Lebel et al., 2012, Treit et al., 2013). This could have long-lasting effects on motor and cognitive functions that rely on cerebellar-thalamocortical pathways, such as motor coordination, learning, and memory. Separately, the SCR is one of the key projection tracts linking the brain stem with deep grey structures with the cerebral cortex, particularly the frontal and parietal lobes, supporting a wide range of cognitive, perceptual and motor systems.

Critically, the present study has succeeded in acquiring high quality neuroimaging in unsedated children, conventionally difficult at this age, and yet important, due to this being one of the most intense periods of brain development in terms of growth, maturation, and potential for neuroplasticity. However, the study was limited by its small sample size, which to some degree was related to participant dropout at follow-up time points, but also challenges with acquiring quality images in children as they are prone to move during scanning. Missing data is a challenging, yet inevitable aspect in most studies of longitudinal nature and with pediatric samples (Lebel et al., 2021). This also impacted our ability to test non-linear associations between PAE and white matter changes over time (Reynolds et al., 2019). Excluding strictly non-complete cases resulted in low overlap of children included for the three time points (Supplementary Fig. 2). Due to unavoidable statistical power limitations, sensitivity analyses on the smaller (overlapping) sample was not feasible. Another potential limitation is the fact that maternal alcohol use during pregnancy was self-reported, which, due to social desirability bias and fear of stigma, may have resulted in under-reporting. Moreover, even though PAE status was confirmed in all cases, for this analysis we were not adequately powered to examine effects of the dosage and frequency of PAE on trajectories of white matter maturation. While we analyzed the effect of PTE in post-hoc sensitivity analyses and found that the effects of PAE-by-time effect for FA in SCP-L were not sensitive to the inclusion of PTE, we do acknowledge that multiple components including the underlying psychosocial risk factors combined with the underpowered sample limit the extent to which independence between PAE and PTE can be exhaustively tested. The impact of these factors, along with other potentially confounding pre- or postnatal exposures which we did not measure or account for, on associations between PAE and white matter maturation should be explored in future studies. Whether children with PAE will continue to show altered patters of white matter microstructural development beyond age 6–7 years should be further explored. Moreover, future investigations should examine the associations between PAE-related white matter microstructural alterations and functional outcomes—the complex nature of this three-point longitudinal trajectory study precluded such an analysis here.

Overall, we describe altered trajectories of white matter maturation between different developmental periods (i.e., neonatal age to age 2–3 versus age 2–3 to 6–7 years), influenced by PAE. Regions identified in this study as showing altered white matter development trajectories in children with PAE have important functional connections to cognitive and motor functions, as well as social and emotional behaviours. This not only emphasizes the importance of acquiring early in-between time points in longitudinal neuroimaging studies of PAE, but also suggests differential sensitive periods of neurodevelopment and vulnerability to environmental exposures, as well as potential optimal windows of early intervention in children with PAE.

## Funding

This work was supported by the Bill and Melinda Gates Foundation [grant number OPP 1017641], National Institute on Alcohol Abuse and Alcoholism [grant numbers R21AA023887, R01AA026834-01], US Brain and Behavior Research Foundation [grant number 24467], Collaborative Initiative on Fetal Alcohol Spectrum Disorders (CIFASD) [grant number U24 AA014811], South African Medical Research Council, UK Government’s Newton Fund [grant number NAF002/1001], Wellcome Trust [grant number 203525/Z/16/Z], South Africa’s National Research Foundation [grant numbers 105865, 120432], ABMRF/The Foundation for Alcohol Research, and Harry Crossley Foundation. The content is solely the responsibility of the authors and does not necessarily represent the official views of the funders.

## CRediT authorship contribution statement

**K.A. Donald:** Conceptualization, Methodology, Writing - original draft, Funding acquisition. **C.J. Hendrikse:** Writing - original draft, data curation. **A. Roos:** Writing – review & editing, Data curation. **C.J. Wedderburn:** Funding acquisition. **S. Subramoney:** Writing – review & editing, Investigation, Data curation. **J.E. Ringshaw:** . **L. Bradford:** Writing – review & editing, Investigation, Data curation. **N. Hoffman:** Writing – review & editing, Project administration, Data curation. **T. Burd:** Writing – review & editing, Project administration, Data curation. **K.L. Narr:** Conceptualization, Writing - review & editing. **R.P. Woods:** Conceptualization, Writing - review & editing. **H.J. Zar:** Conceptualization, Funding, Reveiw and editing. **S.H. Joshi:** Conceptualisation, Funding, analysis, Methodology, Visualization, Review and editing. **D.J. Stein:** Conceptualization, Funding, Reveiw and editing.

## Declaration of competing interest

The authors declare that they have no known competing financial interests or personal relationships that could have appeared to influence the work reported in this paper.

## Data Availability

Data will be made available on request.
