# Supplementary material for: Prenatal alcohol exposure and white matter microstructural changes across the first 6–7 years of life: A longitudinal diffusion tensor imaging study of a South African birth cohort
Source: Neuroimage Clin. 2024 Jan 28;41:103572. doi: 10.1016/j.nicl.2024.103572 (PMC10847766; doi:10.1016/j.nicl.2024.103572)
Supplement: Supplementary data 1 [file mmc1.docx]

# SUPPLEMENTARY MATERIAL

Supplementary figure 1: Flowchart of participation in the main Drakenstein Child Health Study (DCHS) and the current neuroimaging sub-study. *Exclusion criteria provided in main text. **Reasons for unsuccessful scans were: the child did not want to be scanned or was unable to sleep (neonatal and 2–3-year time points) during the scan, or excessive movement during scanning leading to premature termination of scan. ***Reasons for pre- or post-processing exclusion were: processing errors, or poor scan or output quality due to motion or other artifacts.


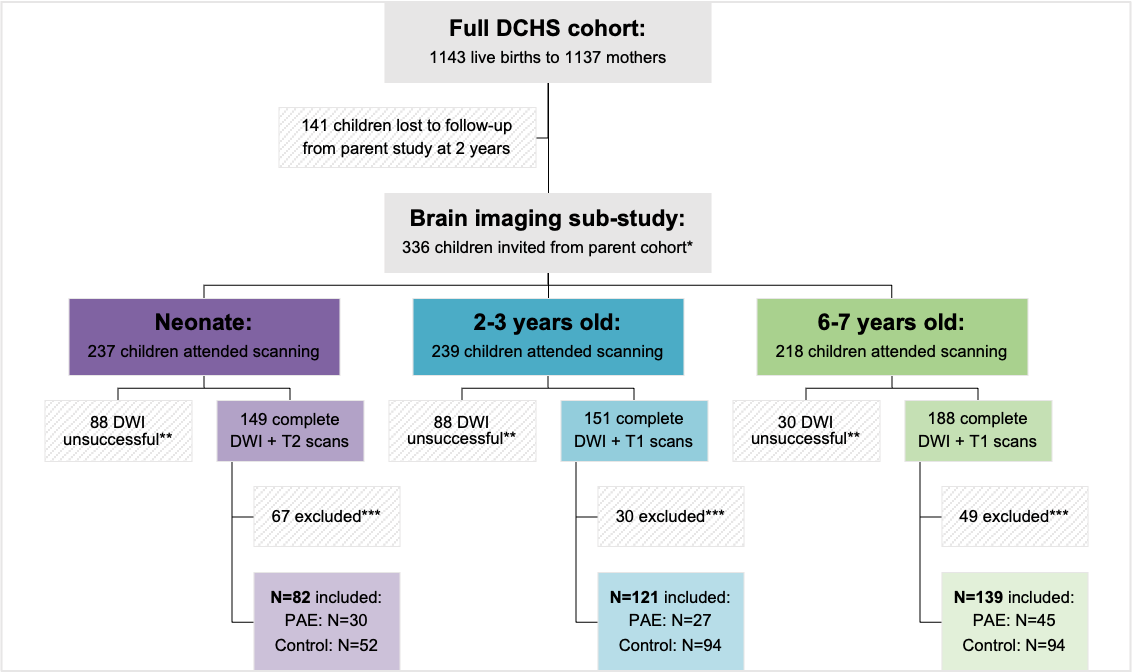


# Supplementary table 1: Longitudinal effects of PAE, Time, and PAE-by-Time interaction on FA and MD.

| **Tract** | **L/R** | **Fractional Anisotropy (FA)^a^** | | | | | | **Mean diffusivity (MD)^a^** | | | | | |
| --- | --- | --- | --- | --- | --- | --- | --- | --- | --- | --- | --- | --- | --- |
|  |  | **PAE** | | **Time** | | **PAE x Time** | | **PAE** | | **Time** | | **PAE x Time** | |
|  |  | **Partial eta^2^** | ***p*** | **Partial eta^2^** | ***p*** | **Partial eta^2^** | ***p*** | **Partial eta^2^** | ***p*** | **Partial eta^2^** | ***p*** | **Partial eta^2^** | ***p*** |
| Superior longitudinal fasciculus | L | 0.016 | 0.025 | 0.842 | <0.001* | 0.002 | 0.733 | 0.001 | 0.656 | 0.962 | <0.001* | 0.019 | 0.055 |
|  | R | 0.009 | 0.143 | 0.864 | <0.001* | 0.027 | 0.046 | 0.016 | 0.031 | 0.951 | <0.001* | 0.022 | 0.038 |
| Sagittal stratum | L | 0.039 | 0.001 | 0.881 | <0.001* | 0.008 | 0.339 | 0.009 | 0.112 | 0.928 | <0.001* | 0.004 | 0.537 |
|  | R | 0.010 | 0.125 | 0.897 | <0.001* | 0.010 | 0.305 | 0.003 | 0.371 | 0.957 | <0.001* | 0.014 | 0.110 |
| Uncinate fasciculus | L | 0.000 | 0.985 | 0.923 | <0.001* | 0.011 | 0.235 | 0.001 | 0.618 | 0.935 | <0.001* | 0.004 | 0.505 |
|  | R | 0.002 | 0.442 | 0.820 | <0.001* | 0.017 | 0.080 | 0.000 | 0.821 | 0.690 | <0.001* | 0.008 | 0.303 |
| Inferior cerebellar peduncle | L | 0.012 | 0.075 | 0.563 | <0.001* | 0.005 | 0.554 | 0.001 | 0.608 | 0.744 | <0.001* | 0.001 | 0.886 |
|  | R | 0.026 | 0.011 | 0.512 | <0.001* | 0.003 | 0.728 | 0.000 | 0.828 | 0.262 | <0.001* | 0.001 | 0.871 |
| Superior cerebellar peduncle | L | 0.023 | 0.007 | 0.522 | <0.001* | 0.042 | 0.001* | 0.008 | 0.126 | 0.109 | <0.001* | 0.035 | 0.008 |
|  | R | 0.025 | 0.021 | 0.784 | <0.001* | 0.019 | 0.125 | 0.016 | 0.108 | 0.477 | <0.001* | 0.013 | 0.373 |
| Corpus callosum genu | n/a | 0.010 | 0.101 | 0.954 | <0.001* | 0.012 | 0.193 | 0.002 | 0.383 | 0.951 | <0.001* | 0.011 | 0.175 |
| Corpus callosum splenium | n/a | 0.007 | 0.225 | 0.924 | <0.001* | 0.001 | 0.876 | 0.001 | 0.607 | 0.978 | <0.001* | 0.008 | 0.408 |
| Fornix | n/a | 0.002 | 0.582 | 0.248 | <0.001* | 0.011 | 0.371 | 0.007 | 0.262 | 0.037 | 0.039 | 0.012 | 0.364 |
| Superior corona radiata | L | 0.005 | 0.248 | 0.926 | <0.001* | 0.016 | 0.131 | 0.008 | 0.136 | 0.968 | <0.001* | 0.019 | 0.059 |
|  | R | 0.010 | 0.118 | 0.924 | <0.001* | 0.003 | 0.670 | 0.007 | 0.146 | 0.965 | <0.001* | 0.021 | 0.047 |

Abbreviations: PAE, prenatal alcohol exposure; L/R, left or right hemisphere; n/a, not applicable.

^a^ Model adjusting for sex and maternal education.

* Survived FDR correction for multiple comparisons.

# Supplementary table 2: Effects of PAE, Time, and PAE-by-time interaction on FA and MD (neonates and 2-3-year-olds).

| **Tract** | **L/R** | **Fractional Anisotropy (FA)^a^** | | | | | | **Mean diffusivity (MD)^a^** | | | | | |
| --- | --- | --- | --- | --- | --- | --- | --- | --- | --- | --- | --- | --- | --- |
|  |  | **PAE** | | **Time** | | **PAE x Time** | | **PAE** | | **Time** | | **PAE x Time** | |
|  |  | **Partial eta^2^** | ***p*** | **Partial eta^2^** | ***p*** | **Partial eta^2^** | ***p*** | **Partial eta^2^** | ***p*** | **Partial eta^2^** | ***p*** | **Partial eta^2^** | ***p*** |
| Superior longitudinal fasciculus | L | 0.055 | 0.007 | 0.372 | <0.001* | 0.003 | 0.531 | 0.006 | 0.265 | 0.917 | <0.001* | 0.004 | 0.372 |
|  | R | 0.036 | 0.015 | 0.818 | <0.001* | 0.000 | 0.929 | 0.025 | 0.027 | 0.929 | <0.001* | 0.006 | 0.283 |
| Sagittal stratum | L | 0.040 | 0.011 | 0.697 | <0.001* | 0.006 | 0.338 | 0.014 | 0.120 | 0.937 | <0.001* | 0.000 | 0.800 |
|  | R | 0.022 | 0.037 | 0.798 | <0.001* | 0.002 | 0.507 | 0.007 | 0.242 | 0.934 | <0.001* | 0.004 | 0.390 |
| Uncinate fasciculus | L | 0.004 | 0.425 | 0.474 | <0.001* | 0.002 | 0.540 | 0.005 | 0.367 | 0.894 | <0.001* | 0.002 | 0.508 |
|  | R | 0.001 | 0.744 | 0.815 | <0.001* | 0.026 | 0.032 | 0.000 | 0.895 | 0.429 | <0.001* | 0.013 | 0.145 |
| Inferior cerebellar peduncle | L | 0.011 | 0.245 | 0.574 | <0.001* | 0.002 | 0.586 | 0.000 | 0.804 | 0.812 | <0.001* | 0.000 | 0.840 |
|  | R | 0.011 | 0.134 | 0.322 | <0.001* | 0.000 | 0.994 | 0.000 | 0.835 | 0.205 | <0.001* | 0.003 | 0.529 |
| Superior cerebellar peduncle | L | 0.029 | 0.019 | 0.252 | <0.001* | 0.043 | 0.004 | 0.028 | 0.039 | 0.131 | <0.001* | 0.006 | 0.341 |
|  | R | 0.025 | 0.026 | 0.400 | <0.001* | 0.021 | 0.039 | 0.028 | 0.126 | 0.530 | <0.001* | 0.017 | 0.230 |
| Corpus callosum genu | n/a | 0.022 | 0.062 | 0.970 | <0.001* | 0.007 | 0.302 | 0.006 | 0.274 | 0.934 | <0.001* | 0.001 | 0.594 |
| Corpus callosum splenium | n/a | 0.010 | 0.187 | 0.838 | <0.001* | 0.001 | 0.714 | 0.005 | 0.352 | 0.964 | <0.001* | 0.002 | 0.552 |
| Fornix | n/a | 0.006 | 0.291 | 0.036 | 0.007 | 0.007 | 0.231 | 0.007 | 0.401 | 0.067 | 0.008 | 0.018 | 0.169 |
| Superior corona radiata | L | 0.018 | 0.057 | 0.922 | <0.001* | 0.000 | 0.996 | 0.016 | 0.078 | 0.959 | <0.001* | 0.006 | 0.291 |
|  | R | 0.016 | 0.109 | 0.886 | <0.001* | 0.000 | 0.884 | 0.014 | 0.101 | 0.946 | <0.001* | 0.007 | 0.261 |

Abbreviations: PAE, prenatal alcohol exposure; L/R, left or right hemisphere; n/a, not applicable.

^a^ Model adjusting for sex and maternal education.

* Survived FDR correction for multiple comparisons.

# Supplementary table 3: Effects of PAE, Time, and PAE-by-time interaction on FA and MD (neonates and 6-7-year-olds).

| **Tract** | **L/R** | **Fractional Anisotropy (FA)^a^** | | | | | | **Mean diffusivity (MD)^a^** | | | | | |
| --- | --- | --- | --- | --- | --- | --- | --- | --- | --- | --- | --- | --- | --- |
|  |  | **PAE** | | **Time** | | **PAE x Time** | | **PAE** | | **Time** | | **PAE x Time** | |
|  |  | **Partial eta^2^** | ***p*** | **Partial eta^2^** | ***p*** | **Partial eta^2^** | ***p*** | **Partial eta^2^** | ***p*** | **Partial eta^2^** | ***p*** | **Partial eta^2^** | ***p*** |
| Superior longitudinal fasciculus | L | 0.014 | 0.086 | 0.822 | <0.001* | 0.002 | 0.530 | 0.003 | 0.432 | 0.969 | <0.001* | 0.005 | 0.357 |
|  | R | 0.004 | 0.430 | 0.895 | <0.001* | 0.021 | 0.078 | 0.011 | 0.118 | 0.944 | <0.001* | 0.022 | 0.028 |
| Sagittal stratum | L | 0.033 | 0.021 | 0.727 | <0.001* | 0.000 | 0.830 | 0.003 | 0.449 | 0.897 | <0.001* | 0.002 | 0.538 |
|  | R | 0.002 | 0.547 | 0.886 | <0.001* | 0.003 | 0.437 | 0.002 | 0.573 | 0.956 | <0.001* | 0.015 | 0.080 |
| Uncinate fasciculus | L | 0.002 | 0.571 | 0.871 | <0.001* | 0.004 | 0.425 | 0.000 | 0.790 | 0.949 | <0.001* | 0.005 | 0.278 |
|  | R | 0.000 | 0.790 | 0.862 | <0.001* | 0.016 | 0.066 | 0.001 | 0.628 | 0.822 | <0.001* | 0.009 | 0.221 |
| Inferior cerebellar peduncle | L | 0.012 | 0.192 | 0.703 | <0.001* | 0.011 | 0.207 | 0.002 | 0.599 | 0.124 | <0.001* | 0.000 | 0.807 |
|  | R | 0.021 | 0.033 | 0.491 | <0.001* | 0.002 | 0.531 | 0.002 | 0.587 | 0.256 | <0.001* | 0.003 | 0.486 |
| Superior cerebellar peduncle | L | 0.000 | 0.896 | 0.631 | <0.001* | 0.002 | 0.524 | 0.000 | 0.967 | 0.050 | <0.001* | 0.030 | 0.010 |
|  | R | 0.006 | 0.354 | 0.826 | <0.001* | 0.005 | 0.388 | 0.006 | 0.397 | 0.155 | <0.001* | 0.002 | 0.620 |
| Corpus callosum genu | n/a | 0.002 | 0.526 | 0.884 | <0.001* | 0.001 | 0.601 | 0.001 | 0.662 | 0.954 | <0.001* | 0.012 | 0.114 |
| Corpus callosum splenium | n/a | 0.011 | 0.237 | 0.940 | <0.001* | 0.001 | 0.680 | 0.000 | 0.788 | 0.973 | <0.001* | 0.003 | 0.471 |
| Fornix | n/a | 0.001 | 0.773 | 0.242 | <0.001* | 0.000 | 0.973 | 0.001 | 0.694 | 0.010 | 0.212 | 0.004 | 0.446 |
| Superior corona radiata | L | 0.001 | 0.690 | 0.923 | <0.001* | 0.010 | 0.165 | 0.016 | 0.078 | 0.959 | <0.001* | 0.006 | 0.291 |
|  | R | 0.008 | 0.246 | 0.937 | <0.001* | 0.003 | 0.486 | 0.005 | 0.292 | 0.963 | <0.001* | 0.022 | 0.033 |

Abbreviations: PAE, prenatal alcohol exposure; L/R, left or right hemisphere; n/a, not applicable.

^a^ Model adjusting for sex and maternal education.

* Survived FDR correction for multiple comparisons.

# Supplementary table 4: Effects of PAE, Time, and PAE-by-time interaction on FA and MD (2-3-year-olds and 6-7-year-olds).

| **Tract** | **L/R** | **Fractional Anisotropy (FA)^a^** | | | | | | **Mean diffusivity (MD)^a^** | | | | | |
| --- | --- | --- | --- | --- | --- | --- | --- | --- | --- | --- | --- | --- | --- |
|  |  | **PAE** | | **Time** | | **PAE x Time** | | **PAE** | | **Time** | | **PAE x Time** | |
|  |  | **Partial eta^2^** | ***p*** | **Partial eta^2^** | ***p*** | **Partial eta^2^** | ***p*** | **Partial eta^2^** | ***p*** | **Partial eta^2^** | ***p*** | **Partial eta^2^** | ***p*** |
| Superior longitudinal fasciculus | L | 0.010 | 0.110 | 0.778 | <0.001* | 0.000 | 0.875 | 0.003 | 0.477 | 0.909 | <0.001* | 0.072 | 0.001* |
|  | R | 0.005 | 0.435 | 0.155 | <0.001* | 0.051 | 0.008 | 0.009 | 0.290 | 0.473 | <0.001* | 0.039 | 0.028 |
| Sagittal stratum | L | 0.054 | <0.001* | 0.907 | <0.001* | 0.009 | 0.171 | 0.015 | 0.102 | 0.538 | <0.001* | 0.009 | 0.199 |
|  | R | 0.013 | 0.177 | 0.544 | <0.001* | 0.022 | 0.086 | 0.000 | 0.913 | 0.803 | <0.001* | 0.057 | 0.010* |
| Uncinate fasciculus | L | 0.000 | 0.913 | 0.938 | <0.001* | 0.013 | 0.103 | 0.000 | 0.877 | 0.674 | <0.001* | 0.002 | 0.513 |
|  | R | 0.015 | 0.083 | 0.024 | <0.001* | 0.001 | 0.663 | 0.005 | 0.303 | 0.374 | <0.001* | 0.002 | 0.525 |
| Inferior cerebellar peduncle | L | 0.021 | 0.042 | 0.101 | <0.001* | 0.001 | 0.678 | 0.001 | 0.682 | 0.783 | <0.001* | 0.002 | 0.540 |
|  | R | 0.048 | 0.010* | 0.139 | <0.001* | 0.002 | 0.645 | 0.002 | 0.654 | 0.034 | 0.049 | 0.002 | 0.647 |
| Superior cerebellar peduncle | L | 0.070 | <0.001* | 0.304 | <0.001* | 0.052 | <0.001* | 0.006 | 0.257 | 0.067 | <0.001* | 0.038 | 0.005* |
|  | R | 0.072 | 0.003* | 0.692 | <0.001* | 0.025 | 0.095 | 0.029 | 0.107 | 0.454 | <0.001* | 0.017 | 0.222 |
| Corpus callosum genu | n/a | 0.015 | 0.129 | 0.931 | <0.001* | 0.026 | 0.048 | 0.000 | 0.842 | 0.132 | <0.001* | 0.029 | 0.030 |
| Corpus callosum splenium | n/a | 0.010 | 0.311 | 0.593 | <0.001* | 0.010 | 0.301 | 0.001 | 0.727 | 0.649 | <0.001* | 0.042 | 0.041 |
| Fornix | n/a | 0.008 | 0.388 | 0.154 | <0.001* | 0.057 | 0.022 | 0.022 | 0.132 | 0.027 | 0.096 | 0.005 | 0.498 |
| Superior corona radiata | L | 0.004 | 0.444 | 0.251 | <0.001* | 0.043 | 0.017 | 0.001 | 0.674 | 0.312 | <0.001* | 0.032 | 0.042 |
|  | R | 0.010 | 0.230 | 0.408 | <0.001* | 0.004 | 0.450 | 0.001 | 0.802 | 0.750 | <0.001* | 0.026 | 0.090 |

Abbreviations: PAE, prenatal alcohol exposure; L/R, left or right hemisphere; n/a, not applicable.

^a^ Model adjusting for sex and maternal education.

* Survived FDR correction for multiple comparisons.

**Supplementary table 5: Longitudinal effects of PAE, Time and PAE-by-Time on FA with and without adjusting for prenatal tobacco exposure.**

|  |  | **Model without PTE^a^** | | | **Model with PTE^b^** | | |
| --- | --- | --- | --- | --- | --- | --- | --- |
| **Tract** | **L/R** | **PAE** | **Time** | **PAE x Time** | **PAE** | **Time** | **PAE x Time** |
| Superior longitudinal fasciculus | L | 0.025 | <0.001* | ns | ns | <0.001* | ns |
|  | R | ns | <0.001* | 0.046 | ns | <0.001* | 0.042 |
| Superior cerebellar peduncle | L | 0.007 | <0.001* | 0.001* | ns | <0.001* | 0.002* |
|  | R | 0.021 | <0.001* | 0.125 | ns | <0.001* | ns |

Abbreviations: PTE, prenatal tobacco exposure; PAE, prenatal alcohol exposure; FA, fractional anisotropy; L/R, left or right hemisphere; ns, not significant at p < 0.05.

^a^ Model adjusting for sex and maternal education.

^b^ Model adjusting for sex, maternal education, and prenatal tobacco exposure.

* Survived FDR correction for multiple comparisons.

**Supplementary figure 2:** Venn diagram showing participant overlap for the three time points.


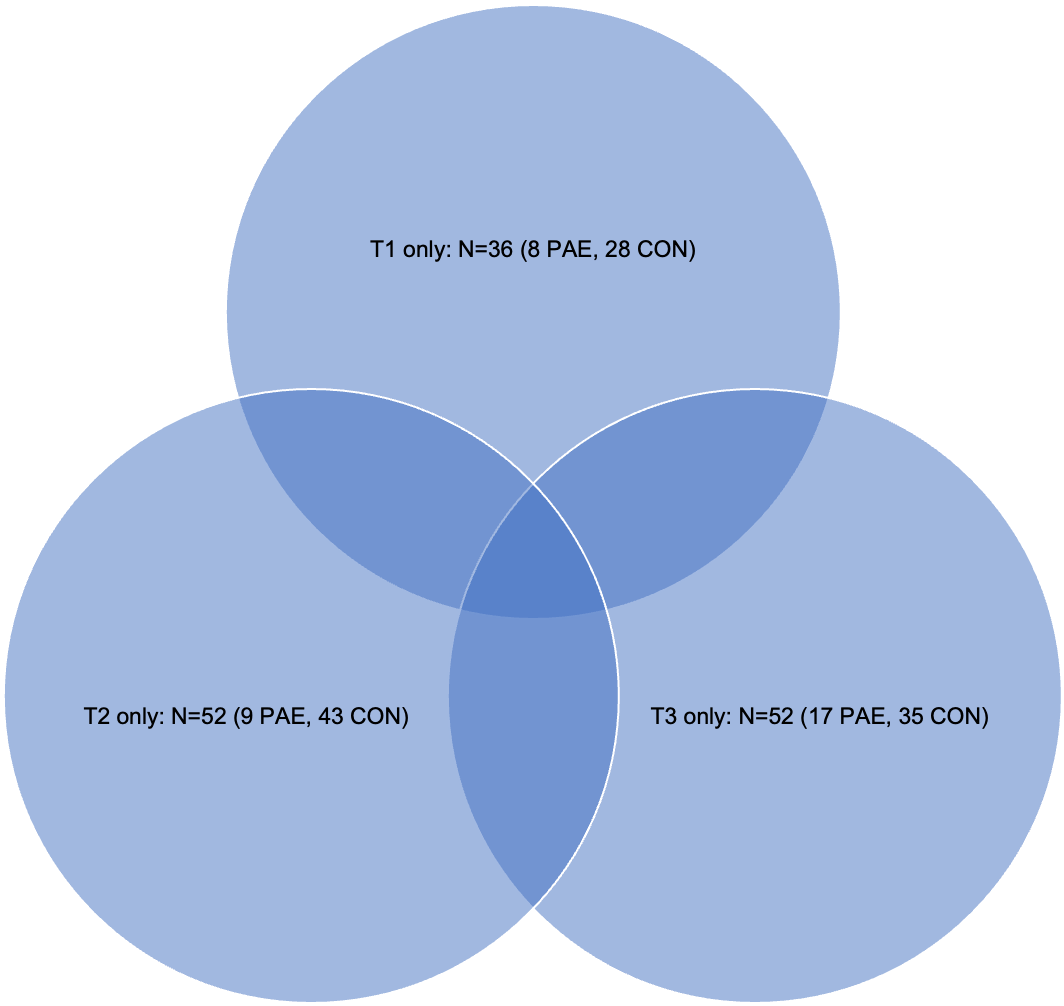


T1+T3: N=28

(15 PAE, 13 CON)

T1+T2: N=10

(5 PAE, 5 CON)

T2+T3: N=51

(11 PAE, 40 CON)

T1+T2+T3:

N=8

(2 PAE,

6 CON)
